# Carbon nanodots as theranostics agents in cancer: advances in design, targeting, and real-time monitoring

**DOI:** 10.1039/d5ra07928d

**Published:** 2026-02-18

**Authors:** Mayank Kumar, Manini Bhatt, Bodhisatwa Das

**Affiliations:** a Department of Biomedical Engineering, Indian Institute of Technology Ropar Punjab India bodhisatwa.das@iitrpr.ac.in

## Abstract

Carbon nanodots (CNDs) have emerged as a promising class of carbon-based nanomaterials for cancer theranostics, uniquely integrating diagnosis and therapy on a single platform. Their ultrasmall size, high aqueous dispersibility, tunable photoluminescence extending into the near-infrared (NIR) window, and compatibility with green, scalable synthesis enable deep-tissue imaging and targeted intervention with reduced systemic toxicity compared with many conventional nanomaterials. This review summarises recent advances in the top-down and bottom-up fabrication of CNDs, including heteroatom doping and surface functionalisation with ligands or stimuli-responsive linkers, and relates these design parameters to optical performance, tumour selectivity, and responsiveness to the tumour microenvironment. Particular emphasis is placed on CND-based platforms for multimodal imaging (fluorescence, MRI, and photoacoustic), controlled-release drug delivery, gene silencing, and light-activated photodynamic and photothermal therapies, as well as emerging synergistic systems that combine these functions for real-time, image-guided treatment. Remaining challenges, such as batch-to-batch variability, incomplete understanding of long-term biosafety (especially for metal-doped CNDs), and limited clinical-scale manufacturing and regulatory readiness, are critically discussed alongside future opportunities, including NIR-II optimisation, protein-corona-resistant surface engineering, and AI-assisted CND design for personalised cancer theranostics.

## Abstract 2

This review focuses on carbon nanodots as a theragnostic probe for multimodal imaging and disease diagnosis, and therapeutic nanoprobe with various application windows.

## Graphical abstract

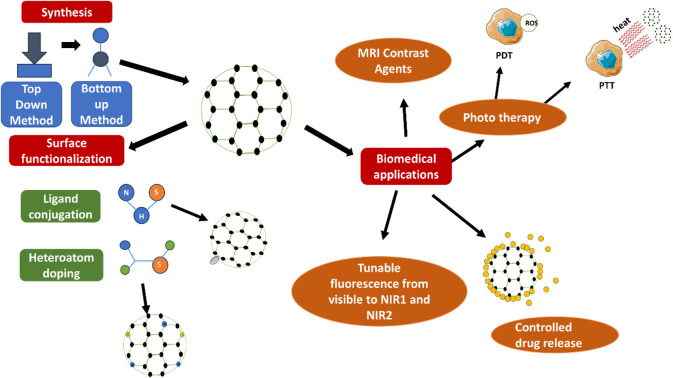

## 1. Introduction

Cancer refers to a group of diseases characterised by abnormal cell signalling in which genetic changes disrupt cellular function and repair. Abnormal cells grow and spread, interfering with normal cells and function. While the treatment of cancer has improved significantly, the disease remains responsible for 8.2 million deaths worldwide each year, and the incidence and fatalities continue to rise worldwide, particularly in low and middle-income countries. Traditional approaches, including surgery, chemotherapy, and radiation, remain primary treatment approaches against cancer. Still, issues such as limited selectivity, systemic toxicity, and therapeutic resistance remain with these modes. The clinical focus has shifted towards theranostics platforms that combine diagnosis and therapy^1^ to enable more precise, timely, and less invasive cancer treatment. These obstacles are being addressed by the advent of theranostics, in which targeted therapy is combined with diagnostic imaging to provide an image-guided, personalised approach to treatment, including real-time monitoring of drug delivery and therapeutic response.^2^ Theranostics applications have been identified for several nanomaterials, including semiconductor quantum dots and polymeric nanoparticles. However, photobleaching, heavy-metal toxicity, and complex production processes are key inhibitory factors to the clinical translation.^3^ Despite the promise of theranostic platforms in addressing the limitations of conventional therapies, their clinical translation remains impeded by inconsistent EPR effects across heterogeneous tumours, regulatory complexities for nanomaterials, and insufficient long-term biocompatibility data. Standardised GMP manufacturing and phase-specific clinical trials demonstrating superior progression-free survival will be essential for broader adoption.

Carbon nanodots (CNDs) are a promising family of next-generation theragnostic agents because they exhibit remarkable photoluminescence, highly efficient water dispersibility, low cytotoxicity, and mild biological interfacing, with a green synthetic process using cost-effective precursors.^4^ In addition, the characteristic features of CNDs, including high quantum yield (QY), negligible photobleaching and blinking, and tunable emission at 660–800 nm, endow them with superior applications in fluorescence imaging and therapeutics such as drug delivery and photodynamic therapy (PDT) and photothermal therapy (PTT) due to facile surface modification enabling easy conjugation with various groups, and facile surface modification.^5,6^ Additionally, their susceptibility to nitrogen or heteroatom doping enhances optical properties and gives responsiveness to tumour-microenvironment cues, enhancing pH-activated imaging and stimuli-responsive drug release.^7^ While CNDs offer compelling advantages over traditional quantum dots, their reported QY values often exhibit batch-to-batch variability and overestimation due to inconsistent characterisation protocols, which limits clinical reproducibility. Standardised absolute QY measurements and machine-learning-based doping optimisation are essential to translate these properties into reliable theranostic platforms (Table 1).

**Table 1:** Comparison of representative carbon-based nanomaterials for cancer theranostics

Carbon-based material	Key composition/features	Diagnostic modality	Therapeutic modality	Performance parameters	Key limitation(s)	Ref.
Carbon nanodots (CNDs)	Quasi-spherical, <10 nm, tunable surface chemistry	FL (visible-NIR), MRI, PA	PDT, PTT, drug delivery	QY up to ∼60%; tumour imaging depth ∼5–10 mm; high photostability	Batch variability; limited long-term toxicity data	8 and 9
Graphene quantum dots (GQDs)	Few-layer graphene fragments	FL, PA	PTT, PDT	High photothermal conversion efficiency (>40%)	Poor biodegradability at larger sizes	10 and 11
Carbon nanotubes (CNTs)	1D tubular carbon structures	PA, Raman	PTT, drug delivery	Strong NIR absorption; deep tissue heating	Bio persistence; toxicity concerns	12 and 13
Fullerene-based nanostructures	C_60_ and derivatives	FL, ROS sensing	PDT	High singlet oxygen yield	Poor water solubility; aggregation	14
Carbon nano shells	Hollow carbon spheres	PA, CT	PTT	High photothermal efficiency; good PA contrast	Larger size limits renal clearance	15 and 16
Biomass-derived CNDs	Green synthesis, biogenic precursors	FL	PDT, drug delivery	Good biocompatibility; low toxicity	Limited emission tunability	17

Recent advances in multiphoton and NIR-I/NIR-II imaging have made it feasible to use CNDs for previously uncommon tissue penetration and resolution. Researchers have shifted emission into the 1000–1700 nm window while increasing quantum yields by over 50% through fine tuning of surface passivation and heteroatom doping (*e.g.*, N, S, P), thereby matching the properties of surface passivation and heteroatom doping (*e.g.*, N, S, P), and matching the properties of heavy-metal quantum dots without their toxicity. According to *in vivo* research, monodisperse NIR-II CNDs can detect invisible microlesions beyond the resolution of traditional imaging by visualising tumours beneath 5 mm of tissue, with signal-to-background ratios greater than 10.^18,19^ These developments highlight CNDs' potential as ultra-sensitive reporters for the real-time identification of deep-tissue tumours and margin evaluation.

In small-animal models, multifunctional CND platforms have produced impressive therapeutic results. Light-activated CNDs coupled to photosensitizers eliminate over 90% of xenografted tumours with minimal collateral damage by achieving simultaneous PDT and PTT ablation with a single low-power laser. By enabling spatiotemporally regulated doxorubicin release, pH- or redox-responsive linkers help to decrease tumour recurrence *in vivo* and overcome multidrug resistance *in vitro*.^20^ In the meantime, siRNA is effectively complexed by the many carboxyl and amine groups on CND surfaces, which shield it from nucleases and deliver gene-silencing payloads that significantly reduce tumour size in mouse models and knock down oncogenes with more than 80% effectiveness.^21^ This review critically assesses recent progress in carbon nanodot-based cancer theragnostics by comparing their diagnostic and therapeutic capabilities with those of other nanomaterial systems, highlighting their distinct merits and limitations. While numerous published reviews examine the synthesis, properties, or specific applications of carbon nanodots (CNDs) in biomedicine, a comprehensive assessment of CNDs' use as integrated cancer theranostic platforms is not widely available. Many reports focus primarily on synthetic methods or separately assess diagnostic and therapeutic applications, without systematically comparing CND design parameters (*e.g.*, size, heteroatom doping, and surface functionalization) with their ability to target, image, or provide therapeutic benefits to patients. Due to advances in near-infrared-I (NIR-I) and near-infrared-II (NIR-II) imaging, tumour microenvironment-responsive systems, and multimodal imaging-guided therapies, there have recently been many new developments in CND-based nanomedicine that warrant an up-to-date synthesis of this growing area of research. Thus, an up-to-date review summarising these advancements and highlighting translational obstacles (*e.g.*, batch-to-batch variability, bio-compatibility issues, and difficulties in scaling for clinical use) is needed as soon as possible.

## 2. Carbon nanodots synthesis and their properties

### 2.1 Bottom-up *vs.* top-down synthesis strategies

The synthesis of CNDs involves both top-down and bottom-up methods, allowing distinct control over electrical structure, surface chemistry, and size. Top-down techniques break down larger carbon precursors (*i.e.*, graphite, carbon nanotubes) into smaller ones of similar or greater strength by laser ablation, arc discharge, or electrochemical oxidation.^22,23^ Approximately 1–5 nm CNDs are produced by arc discharge; however, these must be purified and passivated after synthesis, since they frequently have oxygen-rich surfaces.^24^ Conversely, bottom-up approaches use hydrothermal, microwave, or electrochemical processes to carbonise trash, biomass, or tiny organic compounds. This produces densely functionalized CNDs that are smaller (2–4 nm) and exhibit enhanced optical properties and well-defined surface functionalities.

According to a comparative analysis, top-down CNDs (TD-CNDs) are larger (∼5 nm) and require post-synthetic modification to add chemical groups; however, bottom-up CNDs (BU-CNDs) have smaller average diameters (∼2 nm) and rich C=O/NH surface functionality, as shown in Table 2. Recent comprehensive reviews have systematically classified CND synthesis routes, surface chemistry variations, and their impact on optical/therapeutic properties across biomedical applications.^25^ Furthermore, green synthesis and tunable photoluminescence are combined in electrochemical BU methods using nitriles and ionic liquids to produce ∼3 nm particles, enabling subcellular-resolution fluorescence imaging with a signal-to-background ratio of.^26^ Particle uniformity and scalability are often sacrificed to enable post-synthetic modification and to meet green chemistry considerations, even though both top-down and bottom-up approaches provide precise control over size and surface functionality. To fully realise the potential of CND, future research must evaluate synthesis costs and environmental impacts, optimise green-synthesis purification, and benchmark photoluminescence efficiency.

**Table 2:** Comparative analysis of several CND synthesis methods, emphasising common precursors, particle sizes, surface functions, quantum yield ranges, benefits, and drawbacks of their theragnostic uses in cancer

Method	Precursors	Size (nm)	Surface groups	Quantum yield (QY, %)	Key advantages	Major limitations	References
Top-down method	Graphite, CNDs, GO, carbon soot, carbon fibres, coal, CNTs	5–10	C–O, C–OH	5–20	Direct breakdown, scalable	Requires post- passivation	24
							27
							28
Bottom-up method	Citric acid, biomass, nitriles, glucose, urea, amino acids, sucrose, polyethylene glycol (PEG), lactic acid	2–4	C=O, C–N	10–80+	Tunable PL; green routes	Batch-to-batch variability	26
							29
							30
Electrochemical method	Ionic liquids, nitriles, ethylene glycol, glucose, glycine, hydroquinone	∼3	C=O, NH, –OH	10–50	High PL; no carbon waste	Requires special electrodes	26
							31

### 2.2 Surface chemistry and functionalisation

Surface chemistry modifies a material's surface at the molecular level, thereby altering its physical and chemical characteristics. Its benefits include enhanced colloidal stability, adjustable solubility, and precise addition of reactive functional groups for specific interactions. Surface functionalization is crucial for imparting targeted bioactivity, enhanced quantum yield, and stimulus responsiveness. Covalent (such as amide coupling, esterification, and salinisation) and non-covalent (such as π–π stacking, electrostatic interactions, and coordination) methods are frequently used to modify CND surfaces.^32^ Liu *et al.* demonstrated that B–N-fused anthracenes achieve absorbance >700 nm and 41.8% photothermal efficiency upon nanoparticle encapsulation, highlighting Lewis pair strategies for bandgap narrowing.^33^ Heteroatom doping (N, S, and P) is commonly used when customising electronic states and optical behaviour. For example, nitrogen-doped CNDs exhibit greater photoluminescence quantum yields and increased red-shifted fluorescence.

According to a comprehensive review, surface passivation and doping significantly enhance quantum yield (QY) (by as much as 80%) and add reactive moieties that can be conjugated with polymers, medicinal compounds, or targeted ligands.^34^ Advanced surface engineering strategies enabling precise heteroatom distribution and ligand conjugation for tumour-specific theranostics have recently been elucidated.^35^ Additionally, recent research uses amide coupling to quantify functionalization levels and correlates nitrogen concentration with critical optical transitions.^8^ Despite the potential, achieving uniform functional group density and unchanged heteroatom distribution is not easy, frequently resulting in batch-to-batch variations in quantum yield and bioactivity. Furthermore, significant surface modification may affect long-term stability and biocompatibility or introduce quenching effects. This highlights the need for thorough *in vivo* studies.

### 2.3 Size, structure, and composition

**Fig. 1:**
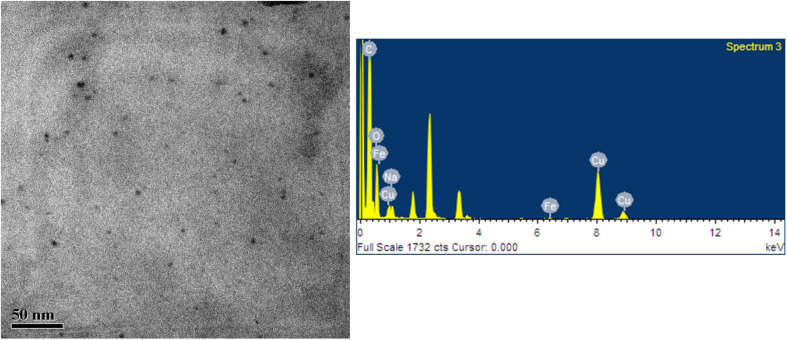
TEM image showing the morphology and size distribution of carbon nanodots (CNDs) and corresponding EDS spectrum confirming their elemental composition (unpublished data of the corresponding author, Dr Bodhisatwa Das).

The size and structural arrangement of CNDs directly affect their theragnostic behaviour and luminescence. Usually ranging in size from 1 to 4 nm (as shown in the TEM image in Fig. 1), bottom-up CNDs have a carbonised polymer core and numerous edge/sp^3^-type defects that carry functional groups containing nitrogen or oxygen. Unless further functionalized, top-down. CNDs, which are bigger (∼5–10 nm) and generated from crystalline graphitic structures, may have simpler surface chemistry.

While TD-CNDs frequently display dominant C–O/C–OH linkages unless post-modified *via* amine or acetone treatments, smaller BU-CNDs are confirmed to exhibit prominent C=O and C–N surface groups by advanced characterisation techniques such as AFM, Raman, XPS, and TEM.^36^ Importantly, the interplay between defect density and the sp^2^/sp^3^ hybridisation ratio controls the formation of localised π-states and mid-gap energy levels that act as key emissive centres. This structural tuning alters surface redox potentials and charge-transfer kinetics, two essential elements for optimising the performance of CNDs in photodynamic therapy and diagnostic imaging, and changes the fluorescence emission wavelength and raises the quantum yield by promoting radiative recombination pathways.

### 2.4 Optical and electronic properties relevant for theragnostics

**Fig. 2:**
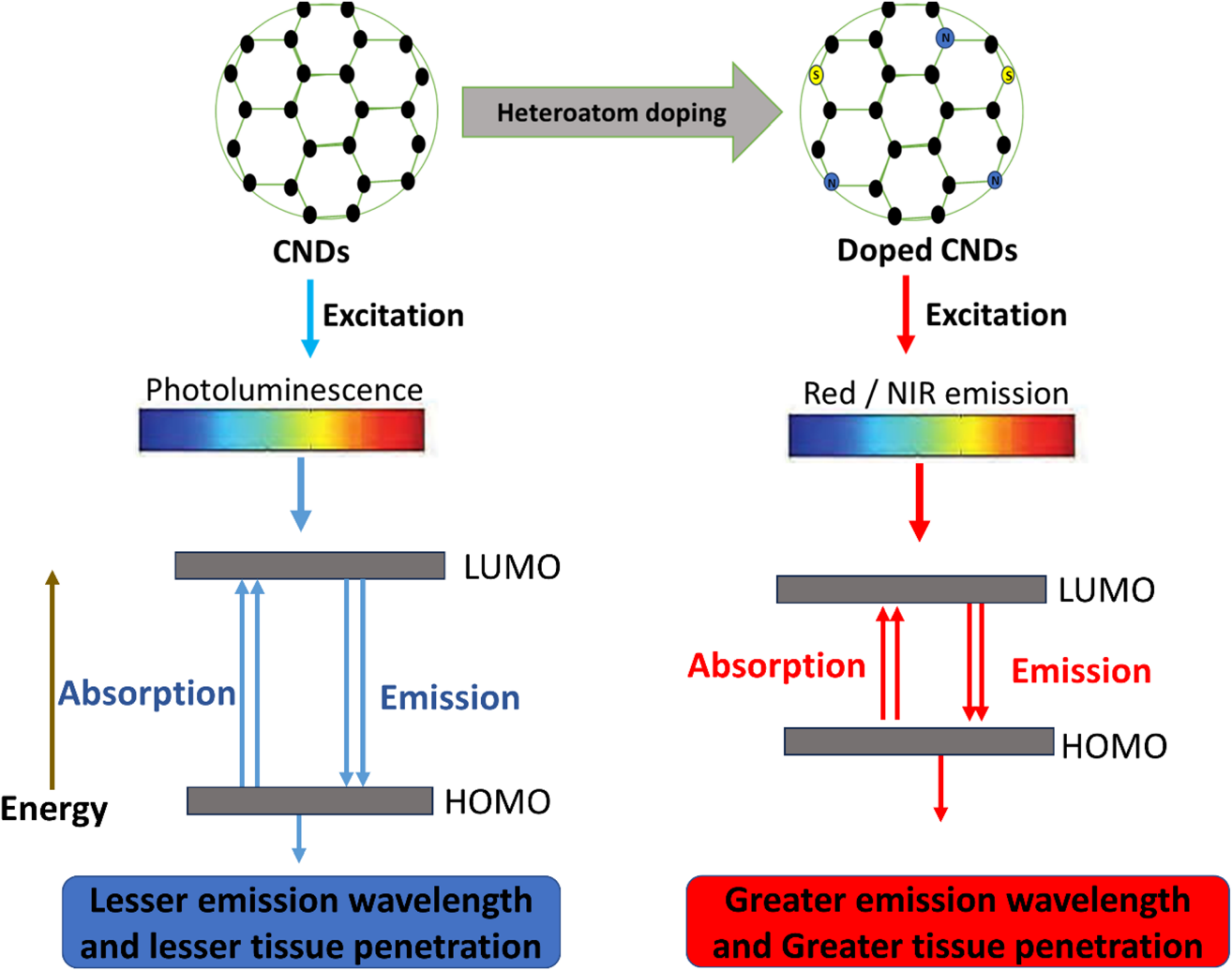
Schematic illustration of photoluminescence in undoped and heteroatom-doped CNDs. In undoped CNDs, excitation produces visible photoluminescence from transitions between the HOMO and LUMO energy levels. This results in shorter wavelengths that penetrate tissues only slightly. Doping with heteroatoms, such as N or S, changes the electronic structure. This allows red or NIR emission from lower-energy transitions. The red-shifted emission helps it penetrate tissues more deeply. This expands the use of CNDs in biomedical imaging and theranostics.

The dual diagnostic and therapeutic functions of CNDs are supported by their optical and electrical characteristics. Quantum confinement (core states) and surface electronic states combine to produce photoluminescence in CNDs; excitation-dependent and independent emissions have been documented. By shifting emission toward the red/NIR window, heteroatom doping (such as N- or S-doping) improves tissue penetration for *in vivo* imaging.^37^ These excitation-dependent PL mechanisms and heteroatom effects on bandgap tuning are well documented across CND variants.^25^ Electronically, CNDs possess redox potentials and catalytic activity similar to those of peroxidase, making them suitable for electrochemical sensing, photoinduced ROS generation, and as components of drug-release or photothermal systems. Due to their adjustable HOMO–LUMO gap (2–4 eV), CNDs make great contrast agents for fluorescence-mediated imaging and photodynamic treatment. B–N fusion dramatically lowers LUMO, enabling NIR absorption while maintaining transparency in the visible range, ideal for selective photothermal activation.^33^ New diamond-like 2D nanodots from carbon nanotubes (CNTs) offer greater structural variation, modified electrical states, and photonic properties for advanced applications.^38^ However, the precise mechanisms underlying CND photoluminescence—whether dominated by quantum confinement or surface states—remain debated, and inconsistent excitation-dependent emission complicates reliable NIR optimisation across batches. Recent 2D nanodot variants show promise but require systematic *in vivo* validation of their enhanced photonic properties against established spherical CNDs to confirm therapeutic superiority (Fig. 2).

## 3. Current treatments for cancer and their limitations

**Fig. 3:**
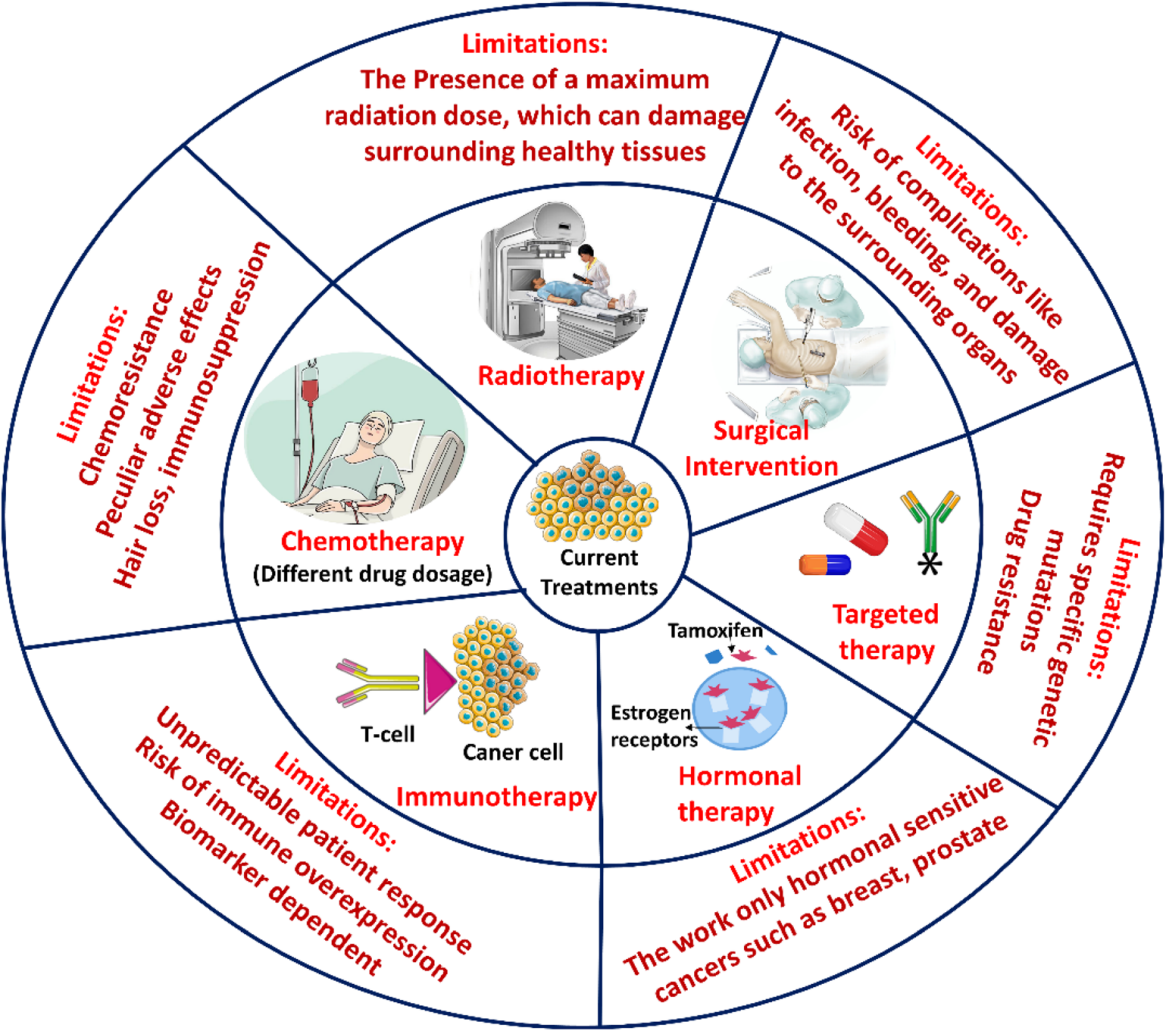
A Schematic illustrates the current state of cancer treatments, including their drawbacks. The difficulties in immunotherapy, hormonal therapy, targeted therapy, radiotherapy, chemotherapy, and surgery are highlighted. Each treatment method is associated with significant clinical restrictions. Non-specific toxicity, medication resistance, restricted tumour type applicability, and immune-related adverse effects are a few of them.

A multimodal approach is used in modern cancer treatment, which includes hormone therapy, radiation, chemotherapy, and surgery (Fig. 3). Although these modalities have improved over the past few decades, increasing therapy alternatives and survival rates, several basic restrictions still exist.

### 3.1 Surgical methods

For local solid tumours, surgical resection is the primary treatment modality. Improvements, including robotically assisted and fluorescence-guided surgery, have lowered collateral damage and increased margins. However, even the most accurate methods often fail to detect microscopic residual disease and occult micro-metastases. Recurrence may be shown by this “minimum residual disease (MRD)”, and perioperative immunosuppression may, ironically, hasten the establishment of metastases.^39^ Recent surgical trials highlight the ongoing challenges in optimising resection and its potential. Shorter hospital stays and higher negative-margin rates resulted from a 25% reduction in operative time, a 30% decrease in intraoperative complications, and a 40% improvement in resection precision, according to a meta-analysis of 25 peer-reviewed studies on AI-enhanced robotic resections across various solid tumours.^40^ In preclinical models, novel intraoperative imaging techniques, such as engineered probiotic bacteria that selectively emit near-infrared fluorescence within hypoxic tumour zones, have demonstrated stable signals for over 72 hours and a five-fold higher tumour-to-background ratio, facilitating the real-time identification of malignant tissue.^41^

Although AI-assisted robots' efficiency and margin-negative improvements have been shown, differences in tumour biology, surgical skill, and institutional resources may limit their practical application. Although novel in preclinical models, the modified probiotic fluorescent technique faces substantial obstacles in obtaining regulatory clearance and evaluating safety before clinical deployment. Therefore, rather than depending on a single technology, integrating precision surgical procedures with customised perioperative immunomodulation will be necessary to sustain MRD-driven recurrence.

Surgery has several drawbacks. It is invasive and has risks, including bleeding, infection, and damage to healthy tissues. It is sometimes challenging to completely remove a tumour, particularly when the disease has progressed or is near vital organs, which increases the likelihood of recurrence. Long recuperation periods and physical and psychological stress are further consequences of surgery.^39^ Furthermore, surgery cannot treat circulating or remaining cancer cells, necessitating additional treatments, and not all patients are suitable for it owing to advanced illness or poor health.

### 3.2 Radiation therapy

Radiation therapy is used clinically to disrupt cancer cells' DNA with high-energy ionising radiation, reducing their ability to divide and grow. It can be administered externally (external beam radiotherapy) or internally, using selectively implanted radioactive sources (brachytherapy), typically in multiple fractions to protect normal tissue. Current external-beam radiotherapy methods, such as IMRT, VAMT, IGRT, and SBRT, and particle treatment (proton and carbon-ion therapy), can deliver high doses of radiation with sub-millimetre precision.^42^ Real-world efficacy to treat cancer is hampered by tumour hypoxia, unnecessary DNA repair responses, and inequitable healthcare access in low-income settings. There are still substantial concerns about dose-limiting toxicities to normal critical organs, which can cause severe toxicities, including fibrosis, organ failure, and secondary malignancies. The SBRT group had comparable biochemical control and significantly less initial gastrointestinal side effects than the fractionated IMRT group, according to recent prospective studies comparing the two treatments for localised prostate cancer.^39^ A recent surgical study for oligometastatic prostate cancer has investigated the use of neoadjuvant radiation and endocrine therapy before robotic-assisted radical prostatectomy. The near top randomised trial demonstrated improved 3-year failure-free survival with manageable toxicity.^43^ Although their submillimetre precision, tumour hypoxia, redundant DNA repair mechanisms, and unequal access worldwide continue to compromise the effectiveness of IMRT, SBRT, and particle therapies, which increase the risk of dose-limiting toxicities, hypo-fractionated SBRT shows promising reductions in acute gastrointestinal side effects, similar to the biochemical control in prostate cancer. However, the lack of long-term outcome data and molecularly guided patient selection emphasises the need to use radiosensitizers and customised hypoxia-DDR biomarkers.

Radiation therapy has certain drawbacks that affect both diseased and healthy cells. The kind of cancer, its stage and location, and the radiation dosage all affect these adverse consequences. It is uncommon for someone to have more than one adverse effect, and some people may not have any. After treatment is finished, most side effects usually disappear. Fatigue is common because the body uses energy to repair healthy cells that may be damaged during treatment. Skin changes can occur in the treated area, such as peeling, redness, or soreness.^44^ There is a rare chance of developing a second cancer from radiation therapy; however, this risk is higher in older individuals or in individuals with some types of cancer.

### 3.3 Chemotherapy

Chemotherapy involves the systemic administration of cytotoxic agents to eradicate rapidly dividing malignant cells. These agents can broadly target multiple tumour types and include alkylating agents, antimetabolites, and anthracyclines. These agents can affect tumour cells in various ways; most disrupt DNA synthesis, while others target the mitotic apparatus. As non-selective agents, they affect normal proliferating tissues, causing side effects such as myelosuppression, mucositis, alopecia, *etc.*^45^ The adverse effects of chemotherapy agents differ by class. Doxorubicin and other anthracyclines have cumulative, dose-dependent cardiotoxicity. Platinum agents, such as cisplatin, can affect the kidneys and hearing. Taxanes, such as paclitaxel, can commonly cause peripheral nerve pain. Vinca alkaloids, such as vincristine, can cause axonal degeneration and nerve damage. Antimetabolites (*e.g.*, 5-fluorouracil) can also cause painful mouth ulcers and diarrhoea.^46^ Tumour cells can develop resistance to multiple medications by increasing DNA repair, preventing cell death, and maximising drug clearance, all of which reduce the effectiveness of treatment.^39^ It suffers from systemic toxicity, limiting the dose that can be given safely, negatively impacting therapy effectiveness, and worsening patients' quality of life.

### 3.4 Hormonal therapy

Hormone therapy is a treatment modality that represents selective targeting in cancer therapy. Some tumours depend on systemic hormones, and hormone therapy alters the tumour's environment. Hormone therapy is a strategy that either reduces hormone production, blocks receptors or receptor binding, or degrades receptors, thereby depriving hormone-driven cancers of the signals to grow. Hormone-sensitive malignancies of the breast, prostate, and gynaecology can experience long-lasting remissions when treated with endocrine drugs. Tamoxifen, aromatase inhibitors, and androgen deprivation therapy are a few of them. Tamoxifen acts by selectively modulating oestrogen receptors.

It binds to the oestrogen receptor (ER)α in breast cancer cells, preventing oestradiol-triggered transcription and proliferation. CYP19A1 (aromatase) inhibitors, such as letrozole, anastrozole, and exemestane, prevent androgens from being converted to oestradiol. This lowers the amount of ligand required for ER activation.^7,47^ GnRH analogues (agonists and antagonists) work on the hypothalamic–pituitary–gonadal axis to inhibit the release of luteinizing hormone and inhibit testosterone production in the testes during androgen deprivation therapy (ADT). In addition to GnRH agents, androgen receptor antagonists such as enzalutamide are used in the ADT modality to inhibit androgen-stimulated gene expression and tumour growth by targeting the androgen receptor (AR) within prostate cancer cells.^48^ Resistance mechanisms can occur by interfering with ligand-independent activation of other growth factor signaling pathways or by mutating receptors.^49^ Additionally, researchers have only started to explore quality of life, metabolism, and bone health under therapy, or overall androgen limitation.^39^ Moreover, hormonal therapy only works well for hormone-sensitive tumours, but it doesn't work for many other forms of cancer.

### 3.5 Immunotherapy

Immunotherapy harnesses the body's immune system to identify and eradicate cancer cells by amplifying natural defence mechanisms or removing inhibitory signals that prevent the immune system from functioning optimally. This transformative strategy encompasses monoclonal antibodies, immune checkpoint inhibitors, cancer vaccines, adoptive cell therapies, and oncolytic viruses, all demonstrating durable and specific anti-tumour responses across haematological malignancies and solid tumours.^9^ Adoptive cell therapies and checkpoint inhibitors (anti-PD-1/PD-L1 and CTLA-4) have improved survival in melanoma, lung, and hematologic tumours. The majority of patients, however, experience no long-term consequences. Pneumonia, endocrinopathies, and colitis are examples of severe immune-mediated toxicities. The shortcomings of predictive biomarkers make patient selection more difficult and increase the risk of overtreatment.^50^ Despite immunotherapy's transformative impact on specific malignancies, its modest response rates of ∼20–30% in solid tumours and the unpredictability of immune-related adverse events underscore the need for patient stratification based on tumour mutational burden and neoantigen load. Integrating CND-based real-time immune monitoring with checkpoint inhibition could enhance precision while mitigating the risks of overtreatment.

### 3.6 Targeted therapy

Targeted therapy for carbon nanodots (CNDs) involves decorating their surfaces with ligands that selectively bind receptors overexpressed on cancer cells. This strategy combines passive drug accumulation *via* the EPR effect and active receptor-mediated uptake. Surface carboxyl, hydroxyl, and amine groups on CNDs allow for covalent coupling of ligands such as folic acid, transferrin, peptides (*e.g.*, RGD), antibodies, and hyaluronic acid *via* carbodiimide or click chemistry. Conjugates introduced into CNDs that are linked to chemotherapeutic drugs, photosensitizers, or nucleic acids could be targeted directly to tumour cells, leading to tumour cell killing *via* photothermal or chemogenetic (chemical induction of apoptosis) mechanisms. Stimuli-responsive linkers, such as pH-sensitive hydrazones or enzyme-cleavable peptides, can release the payload upon arrival in acidic (tumour site) or enzyme-rich (*e.g.*, cathepsin D-rich tumour microenvironment) microenvironments. Tracking biodistribution (real-time optical motion *via* the intrinsic fluorescence of CNDs) and drug release (*via* tracking the intrinsic fluorescence of CNDs) would enhance precision-targeted therapy while minimising off-target toxicity.^51^ There are significant advantages for drugs targeting compact, monoclonal antibodies to oncogenic drivers, such as EGFR, ALK, BRAF, and HER2, in highly defined molecular subgroups, with substantial response rates. However, as with other outliers, there are limits to their selective, comprehensive application; costs and resistance mechanisms can arise through secondary mutations, bypass-induced signalling, and tumour heterogeneity. Targeting emerging clones sequentially often yields only modest increases in survival.^52^ There are landmark studies that demonstrate this strategy. Pardo *et al.* created folic-acid- and transferrin-functionalized CNDs that were loaded with doxorubicin and gemcitabine, demonstrating selective cytotoxicity and fluorescence imaging in folate-receptor-overexpressing cell lines, with minimal effects on regular (healthy) cells.^53^ Prajapati *et al.* present reviews on hyaluronic-acid-conjugated CNDs for CD44 targeting and/or RGD-conjugated CNDs for integrin-binding, all to couple the ligands with photosensitizers to perform fluorescence-guided, receptor-specific photodynamic therapy with excellent tumour selection and reduced systemic spread.^51^ While ligand-functionalized CNDs demonstrate impressive *in vitro* selectivity, their *in vivo* performance is often compromised by protein corona formation, which masks targeting ligands, and by heterogeneous receptor expression across tumour subpopulations. Future designs should incorporate corona-resistant zwitterionic coatings and multi-valent ligand clusters to achieve consistent tumour accumulation beyond EPR limitations.

## 4. CNDs for cancer diagnosis

There are various methods to diagnose cancer, including fluorescence imaging, MRI, multimodal imaging, and targeted molecular imaging with ligand-functionalised CNDs, as outlined in Table 3.

**Table 3:** Overview of diagnostic imaging modalities employing carbon nanodot (CND) variants and functionalization, detailing key performance features, associated challenges and limitations, and key literature references

Diagnostic modality	CND variant/functionalization	Key feature	Challenges and limitations	References
Fluorescence imaging	• Hydrothermal/pyrolysis CNDs (one photon, visible)	• High-resolution subcellular imaging with minimal photobleaching	• Low quantum yield and batch-to-batch variation	54
	• Two-photon CNDs (520 nm)	• Tissue penetration >500 µm	• Require *in vivo* toxicity profiling	55
	• NIR-I (650–900 NM) & NIR-II (1000–1700 nm) CNDs	• Signal to background contrast enhancement in NIR-I	• Need strict production standardisation	
		• Imaging depths >1 cm in NIRII		
MRI & multimodal imaging	• Gd-doped CNDs (*T*_1_ contrast + fluorescence)	• Dual MRI/optical tumour localisation relaxivity >150 mM^−1^ s^−1^ for metastatic	• Potential cytotoxicity and clearance issues of metal dopants	56
	• FeO_4_^−^coordinated CNDs (*T*_2_ contrast)	• Trimodal MRI/fluorescence/photoacoustic mapping	• Maintaining stability and integration of multiple modalities *in vivo*	57
	• Au nanorod-CND assemblies (PA/MR/optical)	• *r*_1_ ∼805 mM^−1^ s^−1^ for metastatic tracking		58
	• Mn-doped CNDs			
Targeted molecular imaging	• Folic acid conjugated CNDs	• Receptor-specific uptake (>5× enhancement)	• Tumour receptor heterogeneity	59
	• EGFR-targeting peptide CNDs	• Sub-nanomolar binding affinity *K*_d_ = 0.8Nm	• Protein corona formation impairs targeting	60
	• Dual- ligand (folate + RGD) CNDs	• Accurate ex vivo tumour imaging	• Requires thought on biodistribution and stability	61
		• Improved *in vivo* selectivity and reduced background		62
Biosensing & early biomarker detector	• Electrochemical CND sensors (CEA)	• Picomolar sensitivity for CEA	High multiplexing complexity	63
	• MiRNA-21 aptamer-CND probes	• 10 fM miRNA-21 detection	Endogenous biomolecule interference need streamlines validation and simplifies assay design	10
	• Enzyme-linked CNDs for MMP activity	• Ratiometric fluorescence response to MMP		64
	• Multiplexed CND arrays	• Simultaneous detection of CEA, AFP, PSA WITH >90% accuracy		65

### 4.1 Fluorescence imaging

Fluorescence imaging is a non-invasive optical method that enables the visualisation and quantification of biological structures and processes by exciting fluorophores at a specific wavelength and detecting emitted photons at longer wavelengths. High-resolution subcellular imaging with minimal photobleaching is enabled by CNDs produced *via* hydrothermal or pyrolytic processes, which exhibit strong one-photon fluorescence in the visible spectrum. They are ideal for intravital imaging of deep-lying tumours because two-photon excitation of these dots, which are centred around 520 nm, under NIR lasers allows tissue penetration depths surpassing 500 µm and minimises photodamage.^54^ Carbon dots engineered to fluoresce in the NIR-I window (650–900 nm) represent a significant advancement because they minimise tissue autofluorescence and scattering, increasing signal-to-background contrast. More recently, NIR-II (1000–1700 nm) fluorescent carbon dots have been employed to achieve imaging depths of over 1 cm, opening the possibility for image-guided surgeries and non-invasive detection of histologically tumour-free regions.^55^ However, the clinical utility of NIR-II CNDs remains constrained by modest quantum yields (<20% in physiological media) and inconsistent emission profiles across synthesis batches, necessitating standardised doping protocols and absolute QY validation. Real-time image-guided surgery applications require further longitudinal studies demonstrating a correlation between fluorescence signal and histopathologic margins in orthotopic tumour models.

CNDs accumulate preferentially in tumours due to enhanced permeability and retention (EPR) effect, or through ligand-mediated active targeting, and their strong NIR-II fluorescence provides real-time, high-contrast delineation of tumour margins for non-destructive diagnostic imaging, *in vivo*, during the time of surgery.^66^ Recent theranostic reviews validate NIR-II CNDs' superior tumour-to-background ratios (>10) and prolonged circulation for image-guided precision therapy.^67^ NIR-I/II CNDs can penetrate deeper tissues for imaging than visible-emitting probes; however, their penetration depth and signal-to-noise ratio are comparable to those of organic fluorophores and generally inferior to those of photoacoustic or MRI-based contrast agents for deep-seated tumours. For this reason, *in vivo* toxicity profiling and strict production standardisation are crucial.

### 4.2 Magnetic resonance imaging (MRI) and multimodal imaging

Magnetic resonance imaging (MRI) is a non-ionising tomographic technique that uses a strong static magnetic field (*B*_0_), radiofrequency (RF) pulses (*B*_1_), and spatially varying magnetic field gradients to produce high-resolution sectional images of the body. The protons within tissues align with the static magnetic field (*B*_0_). Following perturbation of the magnetisation by the RF pulse, the protons return to equilibrium and emit a signal; these signals are collected and reconstructed into slice-by-slice representations of the body's internal economy. MRI machines have built-in post-processing workstations that produce qualitative interpretations and quantitative maps of relaxation parameters. MRI is essential in imaging the neurological, musculoskeletal, cardiovascular, and oncologic populations.^68^*T*_1_-weighted MRI highlights differences in longitudinal (spin–lattice) relaxation times, with tissues that recover their longitudinal magnetisation quickly (short *T*_1_) appearing bright on the image. This contrast mechanism is advantageous for obtaining excellent anatomical detail and delineating fat-rich structures. *T*_1_-weighted imaging can be used to evaluate typical anatomical structures, characterise fat, and characterise post-contrast gadolinium enhancement patterns. *T*_2_-weighted MRI highlights differences in transverse (spin–spin) relaxation times: tissues with a long *T*_2_ value retain their transverse magnetisation longer, so the *T*_2_ signal appears hyperintense. *T*_2_ weighting MRI is particularly sensitive to fluids (*i.e.*, oedema) and is thus great at highlighting pathological processes (*i.e.*, inflammatory changes, cystic lesions, and demyelinating lesions in the central nervous system).^68^ MRI contrast mechanisms lack discussion of critical limitations for oncology applications, including long scan times (>30 min), poor sensitivity to low-concentration contrast agents, and susceptibility to motion artefacts in abdominal imaging. CND-MRI integration requires addressing these gaps through accelerated imaging sequences and high-relaxivity nanoprobes that maintain *T*_1_/*T*_2_ specificity across diverse tumour microenvironments.

Substantial *T*_1_ contrast enhancement and intrinsic fluorescence are combined with gadolinium-doped carbon dots to provide accurate dual-modal MRI/optical imaging for tumour localisation and margin delineation.^56^ In addition to intense visible emission, surface coordination of FeO_4_ nanoparticles onto carbon dots results in *T*_2_ contrast agents with transverse relaxivities >150 mM^−1^ s^−1^, allowing for matching contrast processes.^57^ Using gold's strong photoacoustic signal for deep-tissue vascular mapping, trimodal MRI/fluorescence/photoacoustic probes are produced by incorporating gold nanorods into carbon-dot assemblies.^58^ Dynamic MRI surveillance of metastatic development is enabled by Mn-doped carbon dots, which have a longer circulation half-life and *r*_1_ relaxivity of about 8.5 mM^−1^ s^−1^.^56^ Despite the multimodal adaptability of doped carbon dots, there is no mention of potential cytotoxicity or clearance issues associated with metal doping (*e.g.*, Gd, Mn). Furthermore, one of the biggest obstacles to clinical translation is maintaining the stability and effective integration of many imaging modalities *in vivo*.

### 4.3 Targeted molecular imaging with ligand-functionalised CNDs

For receptor-specific imaging, ligand-functionalized CNDs have been developed. Folic acid-conjugated dots bind specifically to folate receptors overexpressed on various cancer cells, increasing cellular uptake by more than 5-fold and improving *in vivo* tumour contrast.^59^ Peptide-modified carbon dots that target EGFR enable high-contrast fluorescence imaging of carcinoma xenografts and exhibit a sub-nanomolar binding affinity (*K*_d_ = 0.8 nM).^60^*Ex vivo* histological research has confirmed that aptamer-decorated dots targeting prostate-specific membrane antigen (PSMA) provide accurate imaging of prostate tumours with minimal off-target accumulation.^61^ In orthotopic models, dual-ligand strategies that combine folate with RGD peptides further enhance tumour specificity and reduce background signal.^62^ Yet tumour receptor heterogeneity and protein corona formation can undermine the *in vivo* targeting efficacy of ligand-functionalized CNDs, necessitating extensive biodistribution and stability studies.

### 4.4 Biosensing and early cancer biomarkers

Utilising accelerated electron transport at specific electrode interfaces, carbon-dot-enhanced electrochemical systems enable ultra-sensitive, rapid detection of carcinoembryonic antigen at the point of care. Early cancer diagnosis from serum biopsies is possible using fluorescent biosensors composed of aptamer-functionalized carbon dots that detect microRNA-21 at concentrations as low as 10 fM.^10^ Enzyme-linked carbon dot probes (ELCDs), multiplexed carbon dot arrays, and recognition algorithms targeting matrix metalloproteinases (MMPs) have been utilised for real-time imaging of the tumour microenvironment (TME) and display ratiometric fluorescence signals that reflect protease activity.^64^ The multiplexed carbon dot arrays and recognition algorithms have also been used to measure biomarkers (CEA, AFP, PSA) in clinical samples with greater than 90% sensitivity.^65^ Nonetheless, the multiplexity and cross-reactivity of endogenous biomolecules could affect the platforms' robustness and clinical relevance, underscoring the importance of a straightforward, streamlined assay design and validation.

## 5. Tumour targeting strategies and their response to the tumour microenvironment

There are basically two types of targeting strategies, active and passive targeting, which are explained below:

### 5.1 Passive targeting

Passive targeting mechanisms for cancer therapy using carbon nanodots (CNDs) use the enhanced permeability and retention (EPR) effect, allowing preferential accumulation in tumour tissues due to poorly organised vasculature and lymphatic drainage. The EPR effect is a reliable method for selectively targeting solid tumours with CNDs. Nanoparticles with sizes up to 200 nm can extravasate and reside in the tumour interstitium due to the abnormal vasculature of tumours, which has a wide range of large fenestrations (100–800 nm) and a deficiency in lymphatic drainage. This is known as the EPR effect.^11^ Carbon nanodots (CNDs), with an ultra-small core size less than 10 nm and a narrow size distribution, can penetrate further into tumour tissue compared to larger nanocarriers.^69^ The grafting of polyethene glycol (PEG) onto the surface of CNDs prevents protein adsorption, reduces recognition by the mononuclear phagocyte system (MPS), and results in a circulation half-life in hours [rather than minutes]. PEGylated CNDs caused a retention in tumours in mice of three to five times more than unmodified CNDs.^70^ By reducing nonspecific tissue uptake, fine-tuning surface charge achieved through zwitterionic coatings or neutral hydrophilic polymers maximizes EPR-mediated delivery of chemotherapeutics and imaging chemicals.

### 5.2 Active targeting

**Fig. 4:**
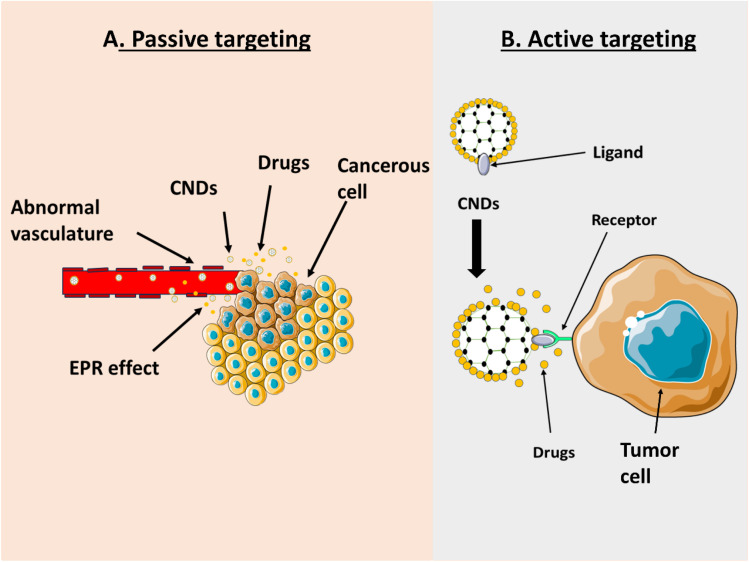
Diagrammatic illustration of carbon nanodots' (CNDs') active and passive targeting strategies for cancer treatment. (A) The increased permeability and retention (EPR) effect passively targets tumour tissues. Here, inadequate lymphatic drainage and leaky blood arteries permit CNDs carrying therapeutic chemicals to congregate near the tumour site. (B) Ligand-functionalized CNDs, which selectively bind to overexpressed receptors on cancer cell membranes, are used for active targeting. This mechanism facilitates localised drug distribution and receptor-mediated endocytosis.

In active targeting, ligands that bind to overexpressed receptors on diseased cells are used to decorate nanocarriers. Ligands are covalently attached to nanoparticle surfaces *via* conjugation chemistries such as EDC/NHS coupling or click reactions, creating a functionalized nanocarrier for cellular targeting, as shown in Fig. 4. Active targeting improves selectivity on CND surfaces. Covalent conjugation strategies, such as copper-free click chemistry or carbodiimide coupling reactions, have been used to attach peptides, aptamers, and antibodies to CNDs without affecting their size and fluorescent properties.^71^ Aptamer-CNDs conjugates have been employed in preclinical studies, enabling surgical resection and fluorescent-guided tumour delineation. Nucleic acid aptamers with strong binding affinity (*K*_d_ 1–10 nM) and low immunogenicity include those targeting nucleolin (AS1411) and sequences specific to the epithelial cell adhesion molecule (EpCAM).^72,73^ To promote receptor-mediated endocytosis, deep tumour penetration, and the intracellular spread of chemotherapeutics, CNDs have been conjugated to short peptide motifs. These include RGD, which binds to integrin αvβ3, and NGR, which interacts with CD13. Resulted in significantly increased cytotoxicity *in vitro* and tumour growth suppression *in vivo*.

### 5.3 Tumour microenvironment-responsive systems

The tumour microenvironment comprises normal, leaky, and abnormal vasculature, resulting in heterogeneous perfusion and hypoxic niches with elevated interstitial fluid pressure. Rapid proliferation and poor blood supply stabilise HIF-1α signalling and promote aerobic glycolysis, which produces lactic acid, lowering extracellular pH to approximately 6.5–6.8. Cancer-associated fibroblasts simultaneously remodel a dense extracellular matrix rich in collagen and hyaluronan. This stiffens the tissues, preventing nanoparticle penetration and immune cell infiltration. An immunosuppressive environment, characterised by regulatory T cells, myeloid-derived suppressor cells, M2-polarised macrophages, and steep nutrient and metabolite gradients, contributes to intratumoral heterogeneity in proliferation, drug susceptibility, and invasive behaviour. This also affects physiological characteristics that challenge therapeutic delivery, highlighting the need for pH-responsive carriers, hypoxia-activated prodrugs, and vessel-normalising agents that can improve therapeutic efficacy.^74^ Zhang *et al.* demonstrated MnOx-mesoporous carbon nanoparticles that enable GSH depletion, Mn^2+^-catalysed OH generation in acidic TME, NIR-enhanced DOX release, and PTT/CDT with 44.2% efficiency.^75^ Tumour cell-targeting and TME-responsive nanoplatforms that integrate multiple responsive mechanisms (pH, redox, enzyme) have shown enhanced precision in preclinical tumour models.^76^ TME-responsive connections have been included in CNDs to provide fine spatiotemporal control over imaging signals and therapeutic activity. An acid-labile hydrazone or imine bond can be used to conjugate chemotherapeutic drugs or fluorescence quenchers *via* pH-sensitive constructs. These linkers rapidly cleave in endosomes or in the extracellular tumour microenvironment at acidic pH (pH 5.0–6.5). This results in drug liberation and on-channel signal activation in a healthy tissue-sparing process.^70^ Multistimulus-responsive designs that combine pH/redox/enzyme sensitivity further enhance spatiotemporal control, as demonstrated in targeted nanoplatforms for solid tumours.^76^ Redox-responsive systems also use disulfide or selenide bridges to link CND cores and payloads; these linkages can be preferentially cleaved by the high levels of glutathione present in cancer cells (2–10 mM), thereby restoring fluorescence while secreting medications intracellularly.^12^ Enzyme-responsive CNDs contain peptide-based substrates unique for matrix metalloproteinases (MMP-2/9) or cathepsin B. When proteases cleave these substrates at the tumour site, imaging and therapeutic functions can be activated, providing controlled, real-time monitoring of enzyme expression and drug release.^13^ Although CND-based TME-responsive systems elegantly exploit acidic pH, redox gradients, and protease activity, most designs are validated in simplified xenograft models that under-represent the spatial and temporal heterogeneity of human tumours. Moving forward, integrating multistimulus logic gates (*e.g.*, pH) and testing in orthotopic, immunocompetent models will be essential to avoid off-target activation and accurately predict clinical performance.

## 6. Advance treatment for cancer by using CNDs

### 6.1 Drug delivery and controlled release systems

As drug delivery vehicles, CNDs have drawn a lot of interest because of their minuscule size, natural biocompatibility, and ease of surface functionalisation. On-demand release and targeted delivery within the acidic TME are made possible by CNDs, which conjugate chemotherapy drugs like doxorubicin or paclitaxel onto their surfaces using pH- or enzyme-sensitive linkers.^77^ The treatment efficacy is improved by this method since it reduces systemic toxicity and increases intra-tumoral drug accumulation.^78^ Debnath *et al.* developed polymer-functionalized CNDs for curcumin delivery that increased A549 lung cancer cell kill by 30% *vs.* free curcumin. The system released ∼80% dose over 72 hours at physiological pH and had 2.5 times the original circulation time in rat models, demonstrating the synergistic advantages of controlled release and prolonged *in vivo* availability.^79^ Engineered doxorubicin-conjugated CNDs through pH-sensitive hydrazone linkers, resulting in 1.6-fold increased cytotoxicity against HeLa cells (IC_50_ 2.5 µg mL^−1^*vs.* 4.0 µg mL^−1^ for free DOX). *In vitro* release studies showed ∼75% drug release at pH 5.5 over 48 hours, compared with ∼30% at pH 7.4, while murine xenograft models demonstrated an increase in circulation half-life from 1.9 hours (free DOX) to 7.3 hours (DOX-CND), leading to approximately a 50% increase in tumour growth inhibition.^80^ Numerous *in vitro* and *in vivo* studies have shown that CND-based carriers are more effective than free medicines. They provide more substantial anticancer effects, manage release rates, and allow longer circulation times.

### 6.2 Photodynamic therapy (PDT)

**Fig. 5:**
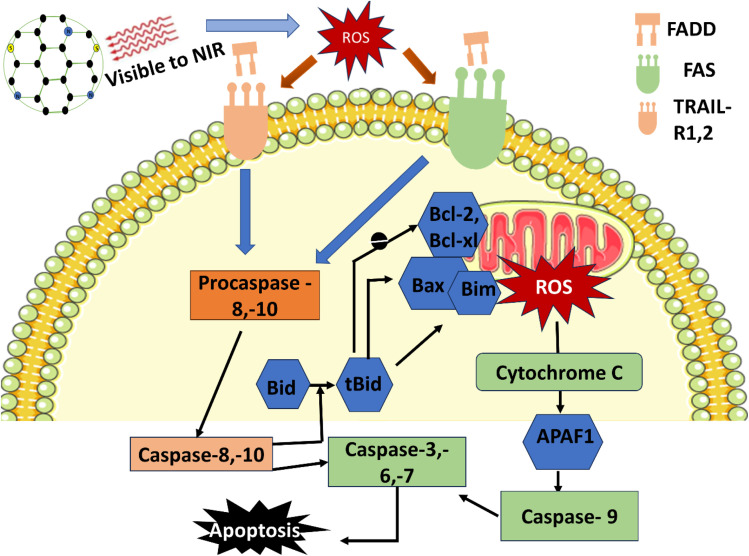
Schematic of carbon nanodot-mediated photodynamic therapy. Carbon nanodots (CNDs) generate reactive oxygen species (ROS) after light exposure *via* Type I and II energy transfer. The ROS generated can initiate the extrinsic (Fas/TRAIL-R-FADD-caspase-8/10) and intrinsic (tBID-Bcl-2 family-cytochrome c-apoptosome-caspase-9) apoptotic pathways, which both converge on the executioner caspases (3, 6, 7) and induce cancer cell death.

Under illumination, photosensitizers generate reactive oxygen species (ROS) that can cause cell death in cancer cells during photodynamic therapy. CNDs take up photons and move from the ground-state singlet state (S_0_) to an excited singlet state (S_1_) before experiencing intersystem crossing to the long-lived triplet (T_1_) state. In the Type II pathway, the triplet CNDs transferred energy to molecular oxygen, producing singlet oxygen. At the same time, they undergo electron transfer reactions in Type I to form superoxide species and hydroxyl radicals. The resulting ROS will oxidatively damage tumour cells' membranes, proteins, and DNA, leading to apoptosis, necrosis, and vascular shutdown 81. CNDs serve as effective scaffolds for photosensitisation due to their excellent photostability and strong visible-to-NIR absorption.^81^ CNDs enhance tumour selectivity and ROS production when coated with aggregation-induced emission dyes, porphyrins, or chlorins. Preclinical models show the potential of CND-PDT systems as next-generation PDT agents by demonstrating deep tissue penetration, minimal off-target damage, and significant cancer reduction.^82,83^ The schematic representation of PDT for cancer cell apoptosis is shown in Fig. 5.

Breast cancer cell death is triggered by carbon nanodot (CND)-mediated photodynamic therapy (PDT) through the production of high levels of reactive oxygen species (ROS) when excited CNDs undergo an irreversible photochemical reaction upon light irradiation. When exposed to light, CNDs enter an excited state, transferring electrons to reach the triplet excited state. CNDs transfer energy to molecular oxygen in this state, generating Type II ROS (singlet oxygen, ^1^O_2_). Additionally, excited CNDs can transfer an electron to molecular oxygen, producing Type I ROS (O_2_˙^−^ and ˙OH). Both Type I and Type II ROS oxidise cellular components directly. Still, they can also activate extrinsic death receptors (Fas, TRAIL-R1/2) that interact with FADD (Fas-associated protein with death domain), mediating the cleavage of procaspase-8/-10. This cleavage activates caspase-8/-10, which processes BID into tBID and translocates it to the mitochondrial membrane. There, tBID promotes activation of Bcl-2 family proteins, thereby increasing mitochondrial outer membrane permeability and cytochrome c release. Cytochrome c binds to Apaf-1, forming the apoptosome, which activates caspase-9. Subsequently, all three caspases (caspase-9, -8, and -10) activate downstream executioner caspases (-3, -6, -7), resulting in DNA fragmentation, membrane blebbing, and programmed cell death in breast cancer cells.

### 6.3 Photothermal therapy (PTT)

To achieve localised hyperthermia, photothermal therapy uses carbon nanodots' (CNDs) high photothermal conversion efficiency and NIR absorption. Upon 808 nm irradiation, surface-doped CNDs undergo rapid nonradiative relaxation. The photon's energy is converted to heat because surface dopants account for 95% of it, raising the tumour temperature above 42 °C. Rapid thermal insult induces protein denaturation, membrane disruption, and mitochondrial dysfunction, ultimately leading to necrosis and apoptosis of cancer cells. Surface functionalization with biomolecules (sulfur- or nitrogen-doped CNDs, PEGylation, or tumour-targeting ligands) may improve tumour accumulation and penetration, enhance biocompatibility, or reduce off-target heating.^84^ CNDs are effective PTT mediators because of their low cytotoxicity and high photothermal conversion efficiency. Sulphur or nitrogen doping of the surface further increases heat production and light absorption. The clinical translation potential of CND-PTT platforms has been highlighted by the rapid temperature elevation (>50 °C), full cancer eradication with low recurrence, and excellent biocompatibility exhibited by CND-based PTT in murine tumour models.^85^ Colloidal stability and biointerface interactions critically determine CND-PTT performance in physiological environments, with surface chemistry optimisation essential for clinical viability.^86^ The effectiveness of CND-mediated PTT was recently confirmed with animal models of breast cancer. Alibrahem *et al.* synthesised sulfur-doped CNDs, which showed a photothermal conversion efficiency of 45% with 1 W cm^−2^ NIR light; a single 5 min irradiation eradicated 90% of the 4T1 tumours with no recurrence after 21 days.^80^ Bopate *et al.* reviewed nitrogen-doped CNDs conjugated with an RGD peptide, resulting in high temperature rises (to 55 °C), and complete tumour ablation, with 3× better survival than free CND controls.^87^ Systematic reviews of CND nanosystems confirm that targeted PTT formulations consistently achieve >40 °C intratumoral temperatures with minimal recurrence in orthotopic models.^88^ Despite these impressive murine outcomes, CND-mediated PTT remains constrained by limited light penetration of 808 nm irradiation in deep-seated or bulky tumours and by heterogeneous heat distribution within the tumour mass. Moreover, most studies rely on short-term toxicity and small animal cohorts; rigorous pharmacokinetic, immunogenicity, and long-term safety evaluations in large-animal or clinically relevant models are still required before genuine translational claims can be made.

### 6.4 Gene delivery and RNA interference

CNDs offer a flexible platform for loading plasmid DNA, microRNA, or small interfering RNA (siRNA) *via* covalent or electrostatic bonding.^89^ Their physical dimensions and surface modifications enable endocytosis, endosomal escape, and long-lasting gene silencing. Suppressed tumour cell proliferation and angiogenesis have resulted from the *in vitro* silencing of oncogenes such as BCL2 and VEGF using CND-siRNA complexes.^90^ Recent advancements provide further evidence of this capability: Luo *et al.* developed hydroxyapatite (HAp-PEI) nanoparticles coated with PEI to deliver KRAS-targeting siRNA to pancreatic cancer cells, achieving efficient gene knockdown and substantial *in vitro* proliferation inhibition with minimal toxicity to healthy pancreatic cells.^91^ Furthermore, while N-doped carbon dots (N-CDs) have been successfully developed for sensitive detection of miR-21—which plays a vital role in many cancers—using ratiometric fluorescence approaches, indicating that CNDs can be highly useful in microRNA-based applications.^92^*In vivo* research validates CNDs as promising gene-delivery and RNA-interference vectors, demonstrating significant tumour growth suppression with low immunogenicity.

## 7. Synergistic theranostics systems using carbon nanodots

Synergistic theragnostic systems leverage the photoluminescent, surface-tunable, and biocompatible properties of carbon nanodots (CDs) to integrate imaging and therapy into a single nanomaterial platform. In these systems, CDs are functionalized with functional groups (–COOH, –OH, –NH_2_) and heteroatom dopants to provide visible- and excitation-tunable fluorescence for real-time optical imaging, while utilizing the local tumour microenvironment (acidic pH and/or a protease-rich environment) to trigger drug release in response to stimuli. These synergetic systems typically take advantage of the enhanced permeability and retention effect for passive targeting, and surface ligands for active targeting of tumour sites. These products also have the benefit of being self-reporting nanocarriers that not only can characterise the biodistribution of drugs and deliver photothermal or photodynamic therapy on demand, but can also report treatment effects by changes in their fluorescence and optical signature.^93^ Jia and colleagues gave an extensive summary describing the engineering of CDs designed for multimodal imaging *via* a combination of fluorescence, photoacoustic, and magnetic resonance contrast (by maximising the proximity of these imaging modalities while also incorporating a therapeutic modality such as photothermal therapy (PTT), photodynamic therapy (PDT), and chemotherapy). They noted that nitrogen-doped CDs could act as efficient singlet-oxygen generators under light irradiation for PDT and as heat converters for PTT, while also being released *via* surface-tethered anticancer drugs in response to tumour acidity. Similarly, in their summary of stimulus-activated systems, enzyme-cleavable linkers on CDs would release a payload only in the tumour microenvironment, jack-knifing jobs such as killing as many tumour cells as possible, while minimising off-target toxicity.^93^ Recent CND theranostic platforms integrating imaging-guided multimodal delivery (PDT/PTT/chemotherapy) show synergistic indices >2.5 across diverse solid tumour models.^88^ Although such multimodal, self-reporting CD platforms are conceptually attractive, their increasing architectural complexity raises substantial challenges for scalable synthesis, batch-to-batch reproducibility, and regulatory approval as combination products. In addition, most synergistic effects have been demonstrated in simplified small-animal models; rigorous head-to-head comparisons with single-modality nanoformulations in orthotopic and immunocompetent tumour models are still needed to justify the added design complexity.

### 7.1 Imaging-guided drug delivery

With their customizable surface chemistry and inherent fluorescence, CNDs are perfect delivery vehicles for imaging-guided medication administration.^94^ Doxorubicin-loaded CNDs are selectively distributed and accumulated in the tumour tissues, and real-time fluorescence imaging has proven valuable for tracking the accumulation of the drug and drug distribution and release profiles.^95^ While receptor-mediated uptake may further be improved through the surface functionalization of tissue-selective ligands such as folate and RGD peptides, there is also an improvement in overall *in vitro* and *in vivo* theranostics efficacy and therapeutic response.^96^ Imaging-guided technologies such as these have a particular advantage for physicians. They can reduce toxicity to off-target tissues and modify dosing schedules based on *in vivo* drug accumulation visibility at the cellular level.

Recent advancements have incorporated stimuli-responsive linkers, such as pH, redox, or enzyme-sensitive bonds, between CNDs and therapeutic payloads to facilitate on-demand drug release.^97^ To validate medication release using a fluorescence “turn-on” signal, Yang *et al.* designed CNDs linked *via* acid-labile hydrazone linkers that release cargo preferentially within acidic endosomes of cancer cells.^97^ This dual role minimises systemic adverse effects and releases active medications exclusively within the TME by ensuring that therapeutic molecules remain dormant during circulation.^98^ Real-time imaging and tailored delivery portend a new era of precision nanomedicines.^99^ Nevertheless, most reported stimuli-responsive CND-drug conjugates are evaluated under idealised *in vitro* conditions that may not faithfully mimic the modest pH and redox gradients present in human tumours. More systematic pharmacokinetic modelling and *in vivo* quantification of “turn-on” signal-drug release correlation are required to ensure that fluorescence truly reports therapeutically relevant payload liberation rather than nonspecific linker degradation.

### 7.2 Dual PDT/PTT systems

Dual PDT and PTT combine ROS-dependent cytotoxicity from visible light with localised hyperthermia (>42 °C) induced by near-infrared (NIR) absorption on a single carbon nanodot (CND) platform. By co-designing the photosensitising and photothermal-converting moieties into CNDs, dual PDT/PTT systems can minimise the light dose required to elicit an effect, overcome hypoxia-induced resistance, and provide synergistic tumour ablation. By utilising complementary mechanisms, combining PDT and PTT on a single CND platform increases anticancer efficacy.^100^ Wang *et al.* developed nitrogen-doped CNDs that generate localised heat under NIR illumination (PTT) and produce reactive oxygen species under visible-light exposure (PDT). These N-CNDs demonstrated exceptional photothermal conversion efficiency (∼31%) and effective tumour cell ablation under dual irradiation.^100^ Similarly, MnOx-mesoporous carbon hybrids achieve PTT/CDT by Mn^2+^ Fenton-like activity amplified by NIR heat (44.2% PCE), GSH consumption, and TME-responsive degradation.^75^ Fe_3_O_4_-carbon composites enable magnetic-PTT synergy, with salt synthesis enhancing porosity for multimodal theranostics.^101^ Fe_3_O_4_ porous carbon exemplifies metal-carbon hybrids for PTT but requires CND-specific Mn/Cu doping optimisation for biocompatibility. *In vitro* studies using HeLa or DU145 cells showed over 90% cell death with combined modulation, indicating synergistic cytotoxicity beyond what single-mode treatment could achieve.^102^ CND theranostic platforms achieving multimodal synergy (PDT/PTT/imaging) with >90% ablation rates have been systematically reviewed, highlighting design principles for clinical translation.^35^ Despite these promising *in vitro* results, dual-irradiation PDT/PTT regimens may face practical constraints *in vivo*, where light penetration, precise spatial control of two wavelengths, and heterogeneous tumour oxygenation can limit the reproducibility of the observed synergy.

Enhancing ROS yield, photothermal efficiency, and NIR absorption depend on surface engineering, including adjusting bandgap structure and surface functional groups.^27^ Regarding antitumour efficacy, in animal models, arginine-functionalized CNDs showed greater targeting capability to tumours, greater depth of tissue, and more effective photothermal treatment than non-modified CNDs.^100^ These multifunctional CNDs may be photoactivated separately or simultaneously, allowing patients to have a more tailored light-based treatment regimen based on appearance, size, and optical access to the tumour.^27^ However, translating the enhanced targeting of arginine-functionalized CNDs from animal models to heterogeneous human tumours requires validation against protein corona effects and intratumoral barriers, which often diminish the efficacy of surface ligands in clinical settings.

### 7.3 Real-time monitoring of therapeutic response

One of its most attractive features is the ability to track the molecular level of therapeutic response in real time. CNDs or nanoprobes that include quencher moieties or sensitive fluorophores that provide “turn-on” signals upon engagement with target analytes can report changes in pH, oxidative stress, or biomarker levels during therapy. For instance, graphene oxide quenchers and FL-labelled substrates (emission ∼450/521 nm) have been used to develop “off-to-on” peptide-based sensors that report caspase-3 activity upon cleavage.^103^ Quantitative monitoring of caspase-3 enzyme activity in living cells is also enabled by ratiometric probes with dual emissions (red *vs.* green channels), such as Ac-Tat-DEVD-CV.^104^ This approach is comparable to dual-emission CNDs that respond to apoptotic markers. Similarly, reversible ratiometric fluorescent probes for glutathione fluctuations have been created to map intracellular thiol dynamics under chemotherapeutic duress. Examples of *in vivo* theranostics models.^105^ These methods support the idea that thiol-reactive or caspase-sensitive nanoprobes can provide immediate feedback on apoptosis activation, guiding dose adjustments and maximising therapeutic time.

### 7.4 Examples of *in vivo* theranostic models

The clinical potential of CD-based theranostics is validated through translational studies conducted in animal models. When folic acid-conjugated CDs loaded with a chemotherapeutic agent were administered to tumour-bearing mice, Xu *et al.* achieved simultaneous tumour imaging and regression, extending survival by 30% compared to controls.^106^ The biocompatibility and safety profile of CDs were highlighted by biodistribution analyses, which showed a preponderance of tumour accumulation and rapid renal clearance. Using both NIR imaging and combined PDT/PTT, Wang *et al.* assessed arginine-doped CDs in a 4T1 breast cancer model. Together with real-time fluorescence monitoring of therapy progress, they saw considerable tumour shrinking (>80%) and minimal harm to healthy tissues. These *in vivo* achievements position CNDs as adaptable agents in tailored theranostics, paving the way for first-in-human trials.^107^ González and Romero studied PEGylated, Gd^3^-doped carbon nanodots surface-modified with folic acid in a CT26 colorectal cancer xenograft model.^27^ The multipurpose CNDs demonstrated receptor-mediated tumour targeting, achieved a five-fold enhancement in *T*_1_-weighted MR contrast, maintained off-target non-toxicity in a first-in-man design, and reduced tumour volume by approximately 75% upon NIR laser irradiation.

### 7.5 Limitations and challenges

While this review shows strong progress so far, significant limitations remain for CNDs to be considered successful for both diagnostic and therapeutic applications. The majority of CND platforms that have been reported in the literature have been evaluated *in vitro* or in subcutaneous xenograft models in mice.^11,53^ The studies provide proof of concept for theranostic effectiveness; however, none of the models are predicting how the tumour will behave based upon human response to cancer due to a lack of accurate environment (tumour heterogeneity and stroma interactions) or true response to drug (immune response, pharmacokinetics) in terms of human cancer. This leads to limited use of experimental results and to an overestimation of drug effectiveness in preclinical studies.

Another major barrier to progress in CND scientific research is reproducibility. As demonstrated in numerous published studies, CNDs are synthesised from a variety of precursor materials *via* different methods and undergo various post-synthetic modifications, ultimately resulting in considerable variability from batch to batch (*i.e.*, particle size, surface chemistry, optical characteristics).^23,43^ While these methods provide synthetic versatility, differences in characterisation procedures impede effective comparison of findings between studies, substantially undermining the validity of established structure–property–function relationships. In addition, variability poses a significant obstacle to scale-up and product release approvals; stability is critical at this stage.

Targeted delivery strategies have shown promising tumour accumulation using ligand-functionalised and tumour-microenvironment-responsive CNDs. While some studies may suggest that *in vivo* targeting efficiency is sufficient, closer examination shows that protein corona formation, along with heterogeneous receptor expression among different tumours, often negatively impacts *in vivo* targeting efficiencies.^88^ In addition, many examples of enhanced tumour uptake have been derived from short-term imaging rather than from quantitative biodistribution or competitive inhibition studies, further reducing confidence in their ability to demonstrate actual receptor-mediated targeting.

Biosafety and long-term fate also remain inadequately addressed. While several studies report minimal short-term toxicity and favourable clearance profiles.^93^ There have been few comprehensive investigations into the long-term biodistribution, immunogenicity, and metabolic degradation of CNDs, especially for metal- and heteroatom-doped CNDs. Studies available to date have been primarily based on small sample sizes and limited measurement durations. Therefore, they cannot provide sufficient information to assess cumulative toxicity or chronic exposure when considering clinical use.

## 8. Conclusion

Carbon nanodots (CNDs) have become a versatile theragnostic agent that bridges oncology diagnostics and therapy due to their ultra-small size, photoluminescent properties, and superior biocompatibility. While heteroatom doping and covalent/non-covalent functionalisation customise the optical characteristics, tumour-targeting potential, and responsiveness to tumour-specific stimuli of CNDs, both top-down and bottom-up production techniques produce CNDs with unique size distributions and surface chemistries. In preclinical studies, CNDs have been shown to provide dual photodynamic and photothermal ablation, pH- or redox-triggered drug release, deep-tissue imaging in the NIR-I/II windows, and efficient gene silencing, achieving >90% tumour reduction with limited off-target effects. Carbon nanodots (CNDs) functionalized with ligands can exploit receptor-mediated endocytosis for selective accumulation within tumours and can be used with multiple imaging modalities (*e.g.*, fluorescence, magnetic resonance imaging (MRI), and ultrasound/photoacoustic imaging) for concomitant, real-time monitoring of therapeutic outcomes. Even with the progress made, several significant hurdles remain, including batch-to-batch reproducibility and consistency in quality; acceptable long-term biosafety of metal-doped variants; reproducible, reliable *in vivo* targeting; addressing protein-corona issues; and achieving therapeutically relevant NIR-II quantum yields.

In the future, research should include a systematic evaluation of biodistribution, clearance pathways, and long-term toxicity of carbon nanodots (CNDs) in clinically relevant animal models, particularly in the context of their demonstrated theranostic applications. To ensure reproducibility and clinical relevance, scalable green synthesis strategies such as biomass-derived precursors, continuous-flow reactors, and adherence to good manufacturing practices are essential to address the batch-to-batch variability highlighted in current CND theranostic systems. Additionally, while protein-corona-resistant hydrophilic shells improve *in vivo* stability, tailored surface chemistries incorporating modular targeting ligands and tumour-microenvironment-responsive linkers are required to achieve consistent tumour selectivity and controlled drug release, as demonstrated by recent CND-based theranostic platforms. Enhancing photophysical properties, particularly red-shifting emission into the NIR-II window (>1000 nm) without sacrificing quantum yield, through defect engineering and supramolecular passivation, will be critical for deep-tissue imaging and image-guided photothermal and photodynamic therapy. Finally, AI-assisted design and data-driven optimisation, combined with advanced imaging and omics analyses, may help rationally correlate CND structure with biological performance, streamlining candidate selection for clinically relevant cancer theranostic applications.

## Author contributions

Mayank Kumar: formal analysis, investigation, writing original draft and editing; Manini Bhatt: conceptualization, original draft and editing; Dr Bodhisatwa Das – conceptualization, funding acquisition, project administration, supervision, writing – review, and editing.

## Conflicts of interest

The authors declare that they have no conflicts of interest.

## Abbreviations

CNDs: Carbon nanodots
PDT: Photodynamic therapy
PTT: Photothermal therapy
NIR: Near-infrared
IMRT: Intensity modulated arc therapy
SBRT: Stereotactic body radiotherapy
VMAT: Volumetric modulation arc therapy
IGRT: Image-guided radiotherapy
PSA: Prostate-specific antigen
CEA: Carcinoembryonic antigen
AFP: Alpha fetoprotein
MRI: Magnetic resonance imaging
ER: Oestrogen receptor
PDL: programmed death ligand
ADT: Androgen deprivation therapy
EPR: Enhanced permeability and retention
TME: Tumour microenvironment
MRD: Minimal residual disease
QY: Quantum yield
TRAIL-R1/2: TNF-related apoptosis-inducing ligand receptor 1 and 2
FADD: Fas-associated death domain protein
BID: BH3-interacting-domain death agonist
BCL-2: B-cell lymphoma 2
BAX: Bcl-2-associated X protein
BIM: Bcl-2 interacting mediator of cell death
APAF-1: Apoptotic protease-activating factor 1

## Data Availability

No primary research results, software or code have been included, and no new data were generated or analyzed as part of the review article. All the figures are also self-drawn by the authors.
